# Mortality of Surgical Operations in the Upper Lake States, Compared with That of Other Regions

**Published:** 1877-01

**Authors:** Edmund Andrews

**Affiliations:** Professor of Princples and Practice of Surgery in Chicago Medical College


### THE MORTALITY OF SURGICAL OPERATIONS IN THE UPPER LAKE STATES, COMPARED WITH THAT OF OTHER REGIONS.

By EDMUND ANDREWS, A.M., M.D.,

Professor of Princples and Practice of Surgery in Chicago Medical College,

Assisted by Thos. B. Lacey, M.D.,

Assistant Surgeon in the National Soldiers' Home.

(Continued.)

### LARYNGOTOMY AND TRACHEOTOMY, ABROAD; FOR DIPHTHERIA OR CROUP.

| AUTHORITIES. | CASES. | DIED. |
|---|---|---|
| Boston City Hosp. Rept. | 9 | 5 |
| Prof. Wilms, of Berlin | 335 | 232 |
| Arch. klin. Chir. Dr. Kühn, B. 8 | 277 | 152 |
| K. k. allg, Krankenhaus, Wien | 10 | 7 |
| St. Bartholomew's Hosp. Repts. | 10 | 6 |
| Totals | 641 | 402 |

Mortality, 63 per cent.

LARYNGOTOMY AND TRACHEOTOMY FOR DIPHTHERIA OR CROUP, ABROAD.

Arranged by Ages, from Prof. Wilms, of Berlin.

| | CASES. | DIED. | PER CENT. MORTALITY. |
|---|---|---|---|
| Under 2 years | 6 | 6 | 100 |
| From 2 to 3 years | 56 | 41 | 73 |
| From 3 to 4 years | 69 | 47 | 68 |
| From 4 to 5 years | 74 | 56 | 76 |
| From 5 to 6 years | 57 | 37 | 65 |
| From 6 to 7 years | 33 | 18 | 55 |
| From 7 to 8 years | 21 | 16 | 76 |
| From 8 to 14 years | 19 | 11 | 58 |

LARYNGOTOMY AND TRACHEOTOMY ABROAD, FOR ŒDEMA GLOTTIDIS.

| AUTHORITIES. | CASES. | DIED. |
|---|---|---|
| Archiv. klin. Chir., B. 8, S. 559 | 73 | 19 |

Mortality, 26 per cent.

LARYNGOTOMY AND TRACHEOTOMY, ABROAD; FOR REMOVAL OF FOREIGN BODIES.

| AUTHORITIES. | CASES. | DIED. |
|---|---|---|
| Archiv. klin. Chir., B. 8, S. 559 | 149 | 40 |
| Dr. Durham, Holmes' Syst. Surg., Vol. II., p. 496 | 167 | 37 |
| Prof. Hamilton, of Columbus Med. Col., O. | 46 | 12 |
| Totals | 362 | 89 |

Mortality 25 per cent.

LARYNGOTOMY AND TRACHEOTOMY FOR ALL CAUSES, ABROAD.

| AUTHORITIES. | CASES. | DIED. |
|---|---|---|
| Boston City Hosp. | 15 | 6 |
| A. E. Durham's collection of cases operated on for foreign bodies, Holmes' Syst. Surg. | 167 | 37 |
| Med. and Surg. Hist. War of Rebel'n, Vol. I., Pt. I. | 20 | 13 |
| K. k. allg. Krankenhaus, Wien | 46 | 22 |
| Statist. des Hôpit. de Paris, 1861–3 | 513 | 354 |
| Hôpit. des Enfants Malades, Paris, 1851–63, quoted by Fischer & Bricheteau, Traitement du Croup, etc. | 1013 | 749 |
| Hôpit. St. Eugénie, Paris, 1854–61, same authority | 396 | 329 |
| Same Hospt., 1862–3. Statist. des Hôpit., Paris | 225 | 153 |
| Other Hosp. of Paris. " " " | 17 | 11 |
| Dr. J. Kühn, Arch. klin. Chir., B. 8, S. 559 | 707 | 269 |
| Totals | 3119 | 1943 |

Mortality, 62 per cent.

OPINIONS OF AUTHORS.

Dr. H. A. Johnson, Prof. of Diseases of the Respiratory and Circulatory Organs in the Chicago Medical College, has had more experience in tracheotomy, probably, than any one in the Lake States. He gives the following opinion:

"Tracheotomy or laryngotomy should be performed in all cases of threatened asphyxia from causes which cannot be speedily removed by other methods, as for instance in cases of

"1. Foreign bodies in the larynx not easily reached and removed through the natural passages.

"2. Œdema of the glottis, threatening death from asphyxia.

"3. Tumors, malignant or non-malignant, in the larynx, threatening asphyxia, and not easily removed through the natural passages.

"4. Acute inflammation, simple or diphtheritic, producing so much obstruction to respiration as to materially diminish oxygenation of the blood.

"The danger in all these cases is not so much from the operation as from the disease for which it is performed, hence the earlier it is done the better.

"The operation is seldom successful in children under two years of age.

"In young subjects especially high tracheotomy is preferable to the low operation.

"Ether may be given when there is not much asphyxia, but in the asphyxiated condition there is already anæsthesia. The exhibition of ether in such condition probably adds to the danger."

A. E. Durham, of Great Britain, (Holmes' System of Surgery,) says laryngotomy should not be performed in early childhood, on account of the small size of the crico-thyroid membrane; nor in acute or extensive disease or injury of the larynx, but is adapted for adults, and especially for males. It is the best operation for foreign bodies impacted in the larynx, and for polypus, stricture and limited chronic disease of the organ. He recommends laryngo-tracheotomy when the patient is too young for laryngotomy, and the surgeon fears to go below, but not for adults, lest the voice be injured.

Tracheotomy he advises for adults, and generally the lower operation in dyspnœa from acute laryngitis, polypi, syphilitic diseases, etc.

A. W. Barclay, of Great Britain, (Holmes' System of Surgery, Vol. IV., p. 513,) says in respect to membranous croup, " Our chief resource for prompt relief to breathing is tracheotomy." He distinguishes membranous croup from diphtheria, and is dubuous about the operation in the latter disease, but concludes that it is justifiable where the dyspnœa is so urgent as to throw other symptoms into the shade.

Erichsen says that tracheotomy and laryngotomy are required in croup and diphtheria when the laryngeal obstruction to respiration is great, and pulmonary and bronchical disease relatively slight. Many cases of dyspnœa from other diseases and from accidents also require it.

Gross advises tracheotomy in urgent dyspnœa in œdema glottidis, diphtheria, etc., and also for foreign bodies in the air passages.

Hamilton prefers crico-thyroid laryngotomy in apnœa from hanging, drowning, and other causes r equiring haste, thyrotomy for laryngeal growths, and high tracheotomy for most cases of diphtheria and croup requiring operative relief. Low tracheotomy he admits only for cases complicated with bronchocele, or for impaction of foreign bodies in the bronchi.

Druitt advises tracheotomy or laryngotomy for threatened asphyxia from croup, diphtheria, or from any other disease.

### CONCLUSIONS.

The opinions of Prof. H. A. Johnson, quoted above, express the results of the best investigations on this subject so correctly that it seems unnecessary to do more than refer to them as in my opinion giving the proper indications for this operation.

As between the Lake States and other regions the figures show to our disadvantage, thus:

Mort. of laryngotomy and tracheotomy, 54 per cent.
Ditto in Lake States, . . . . . . . 81 " "

I think this inferiority in the results of our surgery is due to two causes:

1. Operating on too many patients below the age of two years.

2. Delaying the operation until the patient was too far exhausted to recover.

The operation ought to be performed earlier.

### LITHOTOMY.

Calculous diseases of the urinary organs in the Lake States seem to be less frequent than in Missouri, Kentucky and Tennessee. This is probably due to the fact that the water of the Great Lakes, which furnish the drink of all the towns on its shores is almost destitute of any mineral constituents, and differs but little from rain-water. Hence there is probably no surgeon on these shores who can show a list of cases so numerous as some of the operators elsewhere have done.

### TABLE XIV.

### LITHOTOMY.

| NO. IN MY REC'D | OPERATOR. | Sex. | Age. | DESCRIPTION OF CALCULUS. | COMPLICATIONS. | OPERATION. | Cond. at opr | Time to Operation. | Result. | Time to death or recovery. | Practice. |
|---|---|---|---|---|---|---|---|---|---|---|---|
| ------ | Dr. Z. Pitcher | F | 12 | Large calculus | ------ | Supra-pubic | ------ | ------ | recovered | ------ | Private |
| 54 | Dr. E. Andrews | M | 7 | Calculus fusible, 1 x1½ in. | Dribbling of urine | Lateral | Good | ------ | died | 4 weeks | Hospital |
| 20 | " " | M | 38 | " " 1 x1¾ " | Chronic cystitis | " | " | some years | recovered | ------ | Private |
| 271 | " " | M | 11 | " " 2½x ¾ " | None | " | " | ------ | " | 30 days | " |
| 1294 | " " | M | 5 | " " 1 x1½ " | " | " | " | ------ | " | 6 weeks | " |
| 1720 | " " | F | 55 | " " 1 x1¼ " | " | Forward to pubis & left | " | ------ | " | ------ | " |
| 1746 | " " | M | 65 | " " ¾ " | Severe hemorrhage fr. bladder be. op. | Lateral | Bad | 4 years | died | 2 days | " |
| 1755 | " " | M | 35 | " " ¾ " | None | " | Med | some years | recovered | ------ | " |
| 1766 | " " | M | 6 | " " ¾ " | " | " | Good | 1½ years | " | ------ | " |
| 1783 | " " | M | 35 | " " 1¼x1¾ " | " | " | " | 1½ years | " | 25 days | " |
| 1785 | " " | M | 5 | " " 1¼x1¾ " | " | " | " | ------ | " | ------ | " |
| 1789 | " " | M | 7 | " " 1¼x2 " | " | " | Bad | several yrs. | " | ------ | " |
| 5700 | " " | M | 12 | " " 1 x2 " | Incontinence of ur. | " | Med | 4 years | died | 11 weeks | Hospital |
| 5176 | " " | M | 2 | " " ¾ " | None | " | " | since birth. | recovered | ------ | " |
| 6060 | " " | M | 3 | " " ½ " | " | " | Good | ------ | " | ------ | Private |
| 6151 | " " | M | 35 | " " ⅞ " | " | " | " | some years | " | ------ | " |
| 6550 | " " | M | 52 | 2 Calculi | " | " | Med | " | " | 3 weeks | Hospital |
| 6836 | " " | M | -- | Calculus mulberry, 3½ dr. | " | " | " | ------ | " | 3 " | " |
| 6930 | " " | M | -- | " fusible, 1x1¼ in. | " | " | " | ------ | " | 4 " | " |
| 7017 | " " | M | 40 | 2 " " | " | " | " | ------ | " | 4 " | " |
| 7526 | " " | M | 4 | 2 " small | " | " | Good | ------ | " | ------ | Private |
| 7799 | " " | M | 2 | " 1¼ in. long | " | " | " | ------ | " | 1 mos | " |
| 7693 | " " | M | 5 | Phosphatic calulus, 1x1¼ | " | " | " | 1 year | " | 3 weeks | " |
| 8497 | " " | M | 58 | 9 " " comb. 5½oz | " | " | " | Unknown | " | 6 " | Hospital |
| 8515 | " " | M | 11 | Calculus in memb. portion of uretha, 2½ in. long | " | " | " | " | " | ------ | " |

### Table XIV.—Continued.

| | | | | | | | | | | | |
|---|---|---|---|---|---|---|---|---|---|---|---|
| 8535 | Dr. E. Andrews. | M | 23 | Calculus | None | Lateral | Good | | died | 8 days | Hospital. |
| 8584 | " " | M | 7 | " | " | " | " | 5 years | recovered | | " |
| 7626 | " " | M | 4 | " 1¼x1½ in. | " | " | " | 2 years | " | | Private |
| 7628 | " " | M | 14 | Mulberry calculus, 1 in | " | " | " | some years | " | | " |
| 7721 | " " | M | 35 | Phosphatic " 1⅜x1¾ in. | Delirium and exha. | " | Bad | 2 mos | died | 4 hours | " |
| 8727 | " " | M | 25 | " " 1¼ " | None | " | Good | | recovered | | Hospital. |
| ------ | Dr. J.S.Sherman | M | 7 | Oxalate of lime calculus | " | " | " | 6 mos | " | | " |
| ------ | " " | M | 6 | Small. soft phos. " | " | " | " | | " | | " |
| ------ | " " | M | 40 | Phosphatic " 5 drs. | | " | Med | 4 years | died | | " |
| ------ | " " | M | 78 | Dark-colored " 4 " | | " | Bad | 20 years | " | | " |
| ------ | " " | M | .. | Phosphatic " 3¼ " | | " | Good | 3 years | recovered | | " |
| ------ | " " | M | 35 | Large calculus, 2⅔ oz. | Ulcers and thickening of bladder. Ulcer of rectum. | Bilateral | Bad | 15 years | died | 65 days | " |
| ------ | Cook Co. Hospit | M | 7 | Calculus | | Lithotomy | | | " | | " |
| ------ | Dr. E. Owens | .. | 6 | " | | Lateral | | | recovered | 6 weeks | " |
| ------ | " " | . | 24 | " | | " | Bad | some years | died | 4 " | " |
| ------ | Dr. H. Wardner. | M | 9 | " | None | " | " | 12 mos | recovered | 3 " | Private |
| ------ | Dr. E. D. Kittoe. | M | 42 | 1 large, several small calculi | " | Supra-pubic | Med | 14 years | died | 2 " | " |
| ------ | " " | M | 9 | Calculi both in uretha and bladder | Urinary fistula | Lateral | Bad | 8 years | recovered | | " |
| ------ | " " | M | 7 | Calculus, wt. 6 dr | None | " | Good | 4 years | " | | " |
| ------ | Dr. J. Andrews. | .. | 35 | " | | " | | | " | | " |
| ------ | Dr. E. Powell | M | 7 | " | None | " | Good | | " | | " |
| ------ | Dr. S. S. Bedal | M | 35 | " 2x1½ in | Cystitis | Bilateral | Med | 10 years | " | | " |
| ------ | Dr. J.S.Sherman | .. | 6 | " wt. 3 dr. | None | Lithotomy | | | " | | " |
| ------ | " " | .. | 25 | " ½x1 in. | " | " | Med | | " | | " |

LITHOTRITY.

| | | | | | | | | | | | |
|---|---|---|---|---|---|---|---|---|---|---|---|
| 5366 | Dr. E. Andrews. | M | .. | " concreted around a roll of chewing gum | " | 10 sittings in 19 days | Good | | " | 1 month | Hospital. |

RECAPITULATION.

| | CASES. | DIED. | PER CENT. MORTALITY. |
|---|---|---|---|
| Total Lithotomy | 48 | 11 | 23 |
| Lithot. over age of puberty | 21 | 8 | 38 |
| " under " " | 26 | 3 | 12 |
| Hospital | 21 | 8 | 38 |
| Private practice | 28 | 3 | 11 |
| Lithotrity | 1 | 0 | 0 |

LITHOTOMY ABROAD.

| AUTHORITIES. | CASES. | DIED. |
|---|---|---|
| Gross' cases—Gróss on Ur. Org., p. 276 | 140 | 12 |
| Mott's " " " " p. 276 | 162 | 7 |
| Mettauer's " " " " p. 276 | 91 | 4 |
| Kissam's " " " " p. 276 | 65 | 3 |
| Goldsmith's " " " " p. 276 | 58 | 3 |
| N. R. Smith's " " " " p. 276 | 45 | 3 |
| Dudley's cases, Ky., given by Eve, Trans. Am. Med. As. 1871 | 225 | 7 |
| Eve's cases, Tenn., adults | 51 | 8 |
| " " " children | 45 | 3 |
| Pope's cases, St. Louis, Mo., adults | 35 | 2 |
| " " " " children | 32 | 2 |
| Boston City Hospital Report | 5 | 0 |
| Pennsylvania Hospital | 111 | 90 |
| United States Marine Hospital Report | 1 | 0 |
| Circular No. 3, S. G. O. | 9 | 1 |
| Sir Henry Thompson's British Collection minus, the Norwich cases | 1034 | 123 |
| Mr. Chas. William's table of Norfolk and Norwich cases for 97 years | 1015 | 132 |
| St. Bartholomew's Hospital | 73 | 13 |
| St. George's " | 17 | 1 |
| British Army Reports | 6 | 0 |
| Luneville's Hosp.—Gross on Ur. Org., p. 276 | 365 | 33 |
| Hotel Dieu, La Charitè and Hôp. des Enfants—Gross on Ur. Org., p. 276 | 133 | 33 |
| St. Mary's Hosp., Moscow—Gross on Ur. Org., p. 276 | 411 | 42 |
| Loretto Hosp., Naples— " " " p. 276 | 553 | 82 |
| Saharunpore Disp., India— " " " p. 276 | 824 | 108 |
| K. k. allg. krank. Wien | 65 | 25 |
| Mission Hosp., Canton, China, Dr. Kerr | 187 | 19 |
| Totals | 5758 | 576 |

Mortality, 13 per cent.

The effect of age is shown by the following figures of Sir Henry Thompson:

MORTALITY OF LITHOTOMY AT DIFFERENT AGES.

| DURING THE YEARS. | CASES. | DIED. | PER CENT. MORTALITY. |
|---|---|---|---|
| 1 to 5, inclusive | 473 | 33 | 7 |
| 6 to 11, " | 377 | 16 | 4 |
| 12 to 16, " | 178 | 19 | 11 |
| 17 to 20, " | 76 | 11 | 14 |
| 21 to 29, " | 86 | 11 | 13 |
| 30 to 38, " | 75 | 7 | 9 |
| 39 to 48, " | 100 | 17 | 17 |
| 49 to 58, " | 191 | 40 | 21 |
| 59 to 70, " | 233 | 63 | 27 |
| 71 to 81, " | 38 | 12 | 32 |

The following figures are taken from Mr. Keith's table, British Medical Journal, March 20, 1869, and show the mortality in groups of twenty years:

| AGE. | CASES. | DIED. | PER CENT. MORTALITY. |
|---|---|---|---|
| Under 21 years | 1530 | 151 | 10 |
| 21 to 40 years | 356 | 66 | 19 |
| 41 to 60 " | 477 | 108 | 23 |
| Over 60 " | 479 | 156 | 33 |

Dr. Dulles, of Philadelphia, in the Am. Jour. Med. Sci., July, 1875, gives a table showing how the dangers of lithotomy increase with the weight of the stone, as follows:

| | | | | |
|---|---|---|---|---|
| Under one ounce................ | Mortality, | 9 | per | cent. |
| One to two " ................ | " | 16 | " | " |
| Two to three " ................ | " | 41 | " | " |
| Three to four " ................ | " | 43 | " | " |

Mr. Crosse and Dr. Gardner calculate the mortality according to size as follows for the Norfolk and Norwich Hospital and the Saharupnore Dispensary:

| | CASES. | DIED. |
|---|---|---|
| One ounce and under | 969 | 88 |
| One to two ounces | 249 | 38 |
| Two to three ounces | 68 | 25 |
| Three to four ounces | 21 | 12 |
| Four to five ounces | 11 | 6 |
| Five to six ounces | 7 | 2 |
| Six to seven ounces | 2 | 2 |
| Totals | 1327 | 173 |

MEDIAN OPERATION.

Prof. Gross (Urin. Org., 1876,) gives the following collections:

| | CASES. | DIED. |
|---|---|---|
| American Surgeons | 205 | 9 |
| Reyer, of Cairo | 56 | 9 |
| Norfolk and Norwalk Hosp. | 64 | 13 |
| Pemberton, of Birmingham | 25 | 1 |
| Totals | 350 | 32 |

Mortality, 9 per cent.

BILATERAL OPERATION.

Cases, 536. Died, 41. Mortality, 8 per cent.

RECTO-VESICAL OPERATION.

Cases, 83. Died, 16. Mortality, 19 per cent.

SUPRAPUBIC OPERATION.

Dr. Dulles, of Philadelphia, gives the following comparison between the lateral and suprapubic operrtion, which seems to indicate that tne latter is safest for stones of very large size, but not for those of less than two ounces weight:

| WEIGHT OF STONE. | LATERAL OPER. | | SUPRAPUBIC OPER. | |
|---|---|---|---|---|
| | CASES. | DIED. | CASES. | DIED. |
| Under one ounce | 529 | 47 | 14 | 3 |
| One to two ounces | 119 | 18 | 21 | 4 |
| Two to three ounces | 35 | 16 | 14 | 4 |
| Three to four ounces | 11 | 7 | 19 | 6 |
| Four to five ounces | 5 | 3 | 16 | 7 |
| Five to six ounces | 2 | 0 | 11 | 4 |
| Six to seven ounces | 2 | 2 | 2 | 1 |

LITHOTRITY.

This operation has been inexcusably neglected in the Lake States. I have record of only one case, which was however successful.

LITHOTRITY ABROAD.

| AUTHORITIES. | CASES. | DIED. |
|---|---|---|
| Brodie | 115 | 9 |
| Fergusson | 109 | 12 |
| Keith of Aberdeen | 116 | 7 |
| Thompson | 204 | 13 |
| Crichton | 122 | 8 |
| Boston City Hospital | 1 | 0 |
| Pennsylvania " | 14 | 2 |
| Trans. Am. Med. As., 1871, Prof. Eve | 4 | 0 |
| Trans. N. Y. Med. Soc. | 49 | 9 |
| Statist. Hôp. de Paris, 1861–2–3 | 56 | 9 |
| Civiale, Paris | 591 | 14 |
| K. k. allg. Krank, Wien | 42 | 16 |
| Lücke, Berne | 2 | 0 |
| Dr. J. G. Kerr, Mission Hosp., Canton, China | 30 | 3 |
| Totals | 1455 | 102 |

Mortality, 7 per cent.

The large figures of Civiale, in the above list, showing a mortality only one-third that of the best surgeons elsewhere, have been received with much incredulity, and even gave rise to direct charges of falsehood; especially as the official statistics of the hospitals of his own city show a mortality six times as great.

It would be, perhaps, safer to exclude the Parisian statistics from the list entirely, which would leave the results of the operation elsewhere, as follows: Cases, 808; deaths, 79; mortality, 10 per cent. Probably this is not far from the truth.

OPINIONS OF AUTHORS.

Civiale was almost the inventor of lithotrity, or, at least, he was the first to give it a practical form, and to establish it in the profession. He advocated it warmly as a matter of course.

Sir Henry Thompson says that lithotomy should not be performed in adults, for stones, unless they are above the middle size, say larger than an almond; but lithotrity be substituted for it. Above the middle size he would be guided by the condition of the patient, as to his probable ability to bear the number of sittings requisite to pulverize such large calculi.

Most authors prefer lithotomy for children, both because the risk is slight and because the uretha is inconveniently small for lithotrity, and the child will not readily remain quiet during the sittings of the latter operation; yet Fergusson and others have performed it on children, and Coulsen claims that it will prove safer for them than lithotomy.

Mr. Hawkins (Holmes, Syst. Surg., Vol. IV, p. 1112) opposes lithotrity in children, but that in adults irritable bladders and diseased kidneys do not, as was formerly thought, necessarily forbid it.

Erichsen, Bryant, Morland, Gross, Ashurst and Hamilton agree for the most part as follows.

Lithotomy is generally to be preferred:

1. In children.

2. In very narrow and irritable urethras, with the calculus large.

3. In cases with badly diseased, irritable, sacculated or very atonic bladders.

4. In very hard stones over an inch in diameter, or softer ones over an inch and a half in diameter.

Lithotomy is preferred by these authors in nearly all other cases, but the rules must be subject to exceptions in cases where special combinations of circumstances require it.

CONCLUSIONS.

Lithotomy is a rather rare operation in the Lake States. Its success here is also less than abroad, a fact in striking contrast with most other operations.

Of our forty-eight cases on record eleven died, which is twenty-three per cent., while the rest of the world gives us in over five thousand cases a death rate of only thirteen per cent.

If we compare the Lake States with Missouri, Kentucky and Tennessee the contrast is still greater. In the latter States three hundred and eighty-eight cases give only twenty-two deaths, which is less than six per cent. The only reason which I can offer for our inferior results is, that the disease being rare in the Lake States, the people, though so alert in business affairs, are unaccustomed to think of this disease, and in its earlier stages rarely suspect its existence. On this account they generally neglect it until it is so far advanced that the safest period for operation has passed by.

One of my worst cases was that of a highly educated man who had a calculus for many years, and yet obstinately refused to entertain the idea of its existence, and rejected all the advice of his physician to submit to an examination.

The remarkable results of lithotomy in Missouri, Kentucky and Tennessee are due to several causes:

1. The frequency of the disease keeps the populace alert on the subject, and prompt to seek aid if it is suspected.

2. Owing to the mildness of the climate the houses are extremely open to ventilation, even in many cases to the actual absence of doors and to the leaving out of all "chinking" from the intersticies of the numerous log cabins. Houses are built, not for tightness and warmth as in our cold climate, but for coolness and ventilation. The patients, therefore, are exempt, from many causes of pyæmia and other septic complications.

3. The population is thoroughly well fed and magnificently developed, averaging considerable taller and larger than in most other States. They are, therefore, better subjects for operation than the denizens of northern States, who are largely immigrants from Europe.

4. Dr. Dudley, of Kentucky, selected his cases. Prof. Eve says that in addition to his two hundred and twenty-five operations there were about eighteen patients, or seven per cent. of all whom Dudley rejected on account of their bad condition. If any surgeon rejects seven per cent. of his most unpromising cases it will make a great difference in the per cent. of mortality, yet few conscientious men will feel justified in refusing to give a man a chance for his life simply because that chance is not as good as the average.

Dr. Dudley, I believe, generally used a gorget, and the bilateral incision. If I am correct in this, his large number of selected cases is probably the reason why our figures show only eight per cent. of mortality for the bilateral method. The old rule is probably true which reserves the bilateral incision for the larger stones.

The results of the median operation seem to show very favorably, giving a mortality of only nine per cent., but it must be remembered that these are selected cases, only small stones being operated on by that method, and hence the figures give no true basis of comparison. Mr. A. Poland, in Holmes' System of Surgery, compares sixty-four cases of median with sixty-four cases of lateral lithotomy, and finds that the lateral proved the safest. On the whole it seems doubtful whether the median method possesses any decided advantage, and in future we shall hear less of it except in children, because it is now conceded that the majority of adult cases adapted to that plan are better treated by lithotrity.

Suprapubic lithotomy seems, if the small number of tabulated cases can be trusted, to be the safest plan in stones weighing over two ounces.

### LITHOTRITY.

This operation has been greatly and improperly neglected among us. We have not cases enough to determine its risk. Abroad the average mortality has been seven per cent., or if we exclude the enormous and disputed list of Civiale, it will be ten per cent.

The later operations seem more successful than the earlier. Perhaps seven per cent. may approximately represent the present average.

There is no doubt that in almost all adult cases lithotrity is the safest operation, and that it should be preferred whenever special conditions of the patient do not render it ineligible. The present drift of science favors extending the application of the operation as much as possible.

### OPERATIONS FOR MECHANICAL OBSTRUCTIONS OF THE INTESTINES.

Of these I find record of fifty cases, which are here subjoined:

### TABLE XV.

### OPERATIONS FOR MECHANICAL OBSTRUCTION OF INTESTINES.

| No. in Rec | OPERATOR OR REPORTER. | Age. | CAUSE OF OPERATION. | COMPLICATIONS. | OPERATION. | Con-diti'n | Duration before operation | Result. | Practice. |
|---|---|---|---|---|---|---|---|---|---|
| 22 | Dr. E. Andrews | 14 | Strangulated congenital hernia | None | Herniotomy | Good | | Recov'r'd | Private |
| 21 | " " | 60 | " scrotal " | " | " | " | | " | " |
| 695 | " " | 40 | " femoral " | " | " | " | | " | " |
| 1562 | " " | 58 | " umbilical " | Mortified intestine | " | " | | " | " |
| 7694 | " " | 45 | " femoral " | None | " | " | 36 hours | " | " |
| 7516 | " " | 38 | " " " | " | " | " | | " | " |
| 7722 | " " | 50 | " " " | " | " opened sac | Med | 3 days | " | " |
| 8770 | " " | 30 | " inguinal " | " | " " " | Good | 50 hours | " | Hospital. |
| 7725 | " " | 4 | " " " | " | " | " | 30 hours | " | Private |
| 7756 | " " | 35 | " " " | Gut mortified | " artificial anus | Bad | 6 days | Died | " |
| 7754 | " " | 30 | " " " | None | " sac opened | Good | 5 days | Recov'r'd | " |
| 8770 | " " | 27 | " " " | " | " " " | Med. | 48 hours | " | Hospital. |
| ---- | Dr. A. Fisher | 60 | " " " | Mortified intestine | " artificial anus | Bad | 3 days | " | " |
| ---- | " " | 65 | " " " | None | " | " | 20 hours | " | " |
| ---- | " " | 20 | " " " | " | " | Good | 36 " | " | " |
| ---- | " " | 36 | " " " | " | " | Bad | 40 " | " | " |
| ---- | Dr. F. W. Mercer | -- | " hernia | | " | | 10 " | " | " |
| ---- | Cook Co. Hospit. | -- | " " | | " | | | " | " |
| ---- | Dr. H. A. Johnson | - | " femoral hernia | | " | | | Died | Private |
| ---- | Dr. N. Senn | 60 | " inguinal " | Peritonitis | " opened sac | Bad | 4 days | " | " |
| ---- | " " | 65 | " femoral " | " | " " " | " | 6 " | " | " |
| ---- | " " | 50 | " " " | None | " " " | " | 6 " | Recov'r'd | " |
| ---- | " " | 52 | " inguinal " | Gut mortified | " " " | Med. | 5 " | Died | " |
| ---- | " " | 62 | " " " | " " | " " " | " | 4 " | " | " |
| ---- | " " | 28 | " " " | " " | " " " | " | 2 " | Recov'r'd | " |
| ---- | Dr. J. Andrews | 51 | " femoral " | | " | | 2 " | " | " |
| ---- | " " | 84 | " scrotal " | | " | | 15 hours | " | " |
| ---- | " " | 48 | " " " | Gut mortified | " | | 10 " | Died | " |
| ---- | " " | 34 | " inguinal " | " " | " | | 2 days | Recov'r'd | " |
| ---- | " " | 55 | " " " | Cartilaginous tumor on protud. int. | " | | 2 or 3 day | Died | " |
| ---- | Dr. E. W. Lee | -- | " " "(large) | None | Pneumatic aspiration | Bad | 5 hours | Recov'r'd | " |
| ---- | Dr. F. W. Mercer | -- | " hernia | Not stated | Herniotomy | | | " | |
| ---- | " " | -- | " " | " " | " | | | " | |
| ---- | " " | -- | " " | " " | " | | | " | |
| 7787 | " " | 55 | " inguinal hernia | None | " opened sac | Good | 4 hours | " | Private. |

### Table XV.—Continued.

| | | | | | | | | | |
|---|---|---|---|---|---|---|---|---|---|
| ---- | Dr. E. Andrews.. | .. | Cancerous stricture of rectum | None | Lumbar colotomy | Med.. | | Recov'r'd | Hospital. |
| 92 | " " .. | 25 | " " " | | Forced dilatation | Bad.. | 2 years... | Improved | Private.. |
| 133 | " " .. | .. | Stricture of rectum | None | Gradual " | Good | | " | " .. |
| 6232 | " " .. | 36 | Cancerous stricture of rectum | | Forced " | " .. | 2 years... | " | " .. |
| 6596 | " " .. | 43 | Stricture of rectum | None | " " | " .. | 2 " ... | " | Hospital. |
| 7227 | " " .. | .. | " " | | Cut the stricture | " .. | | Cured ... | " |
| 7602 | " " .. | 30 | " " | Lupus of anus | Forced dilatation | " .. | | Improved | Private.. |
| ---- | " " .. | 60 | " " | | " " | Bad.. | | Died .... | Hospital. |
| ---- | " " .. | 28 | " " | None | " " | Good | | Improved | " |
| 7784 | " " .. | 35 | " " | " | Gradual " | " .. | | " | Private.. |
| ---- | " " .. | 35 | Intussusception | " | Pneumatic aspiration | Med.. | 3 days ... | Died .... | " .. |
| ---- | " " .. | 25 | " | " | Forced injections | " .. | 1 day..... | " | Hospital. |
| ---- | " " .. | 20 | " | " | " " | Good | 8 hours .. | Cured ... | Private.. |
| ---- | " " .. | 37 | " | " | " " | " .. | 24 hours . | " | " .. |
| ---- | " " .. | 37 | " | Gut mortified | " " | Bad.. | | Died .... | " .. |
| ---- | " " .. | 40 | " | None | " " | Med . | 1 day..... | " | " .. |

RECAPITULATION.

| | CASES. | DIED. | PER CENT. MORTALITY |
|---|---|---|---|
| Herniotomy | 34 | 8 | 24 |
| Pneumatic aspiration of strangulated hernia | 1 | 0 | 0 |
| Forced injections for intussusception | 5 | 3 | 60 |
| Pneumatic aspiration for intussusception | 1 | 1 | 100 |
| Forced dilatation of stricture of rectum | 6 | 1 | 17 |
| Gradual dilatation of stricture of rectum | 2 | 0 | 0 |
| Incision of stricture of rectum | 1 | 0 | 0 |

HERNIOTOMY ABROAD.

The literature of the profession furnish the following statistics:

| AUTHORITIES. | CASES. | DIED. |
|---|---|---|
| Bellevue and Charity Hosp., New York, Hamilton | 31 | 15 |
| Boston City Hosp | 10 | 7 |
| Boston Private Practice of Dr. Cheever | 17 | 7 |
| United States Marine Hosp. Repts | 5 | 1 |
| London Hosps.: quoted Arch. klin. Chir., Bd. 8, S. 30 | 326 | 136 |
| Large British Prov. Hosps. " " " " " | 177 | 72 |
| Small " " " " " " " " | 118 | 53 |
| Dutrepont's personal observations | 12 | 1 |
| Paris Hosps., Old statistics of Malgaigne | 220 | 133 |
| " " Statist. des Hôp. de Paris, 1861–2–3 | 172 | 136 |
| Textor's Cases, Wurtzburg | 56 | 24 |
| K. k. allg. Krankenhaus, Wien | 259 | 114 |
| Deutsch, Zeitschrift, Bd. 2, S. 381 | 27 | 16 |
| Arch. klin. Chir., Bd. 11, S. 320, 341 | 45 | 15 |
| Totals | 1475 | 730 |

Mortality abroad, 49 per cent. Mortality in the Lake States, 24 per cent.

It thus appears that the danger of this operation in the Lake States is less than half that of the published statistics abroad. I can only account for this by the fact that the alert, wide awake western man when he has a strangulated hernia which he cannot reduce himself, comprehends the urgency of his case, and promptly sends for professional help. The operations are therefore performed early, and are consequently successful.

The slowness of the same people to apply for help in cases of calculus is because the latter disease being rare here, and coming on insiduously, is not understood nor suspected until a late period of its progress.

### OPINIONS OF AUTHORS.

There is no controversy, of course, as to the frequent necessity of this operation, and still further, all surgeons are agreed that when necessary it should be performed at the earliest practicable moment, as every hour increases its danger.

Almost the only point of controversy has been whether the peritoneal sac should be opened, or the stricture divided outside the sac.

Erichsen, Hey, Ashton, Key, Luke, Druitt, Bryant and Holmes prefer division of the stricture outside the sac except where fear of mortified intestine or other special reasons forbid. Sir Astley Cooper preferred the extraperitoneal division in large old hernias, operated on early. Ashurst favors it in all cases where the taxis is justifiable. Gross and Birkett favor it in mild and recent cases, while Gant, Lawrence, J. F. Smith, Hamilton and Pirrie think that the sac should usually be opened, and the extraperitoneal division be reserved for exceptionally favorable cases.

Statistics have been gathered to decide the question, but I have not inserted them, because they are worthless. At first glance the extraperitoneal shows much less mortality than the other method; but the fact that the early cases only are selected for extraperitoneal division, shows that this operation is performed on much the safest class of patients, while the later cases, where there is risk that the gut may be mortified, compel the opening of the sac.

Late cases are always dangerous, whether there is mortification or not; hence, there is no proper basis of comparison. There are no reliable statistics of the two operations performed on patients of the same quality.

### CONCLUSIONS.

Herniotomy is indicated whenever other means of relief fail, and should not be delayed a single hour unnecessarily. When there is strong reason to fear that the gut may be already mortified the taxis should be omitted for fear of returning a mortified intestine, and herniotomy should be resorted to at once. Every hour of delay increases the danger.

If the strangulation is so recent and mild that it is morally certain that no mortification has yet occurred, the extraperitoneal division is the best, unless special circumstances forbid, for it diminishes the risk of peritonitis. The aspirator, of course, should never be used in a case deemed to be too far advanced toward mortification for prudent taxis, but it seems probable that in early stages it may be a valuable assistant to successful reduction, and experience has not yet developed any special dangers in its use.

(To be Continued.)

